# Image Quality Control in Lumbar Spine Radiography Using Enhanced U-Net Neural Networks

**DOI:** 10.3389/fpubh.2022.891766

**Published:** 2022-04-26

**Authors:** Xiao Chen, Qingshan Deng, Qiang Wang, Xinmiao Liu, Lei Chen, Jinjin Liu, Shuangquan Li, Meihao Wang, Guoquan Cao

**Affiliations:** ^1^Department of Radiology, Key Laboratory of Intelligent Medical Imaging of Wenzhou, First Affiliated Hospital of Wenzhou Medical University, Wenzhou, China; ^2^Shanghai United Imaging Intelligence Co., Ltd., Shanghai, China; ^3^School of Laboratory Medicine and Life Sciences, Wenzhou Medical University, Wenzhou, China

**Keywords:** deep learning, quality control, U-net, medical imaging, radiography, image segmentation

## Abstract

**Purpose:**

To standardize the radiography imaging procedure, an image quality control framework using the deep learning technique was developed to segment and evaluate lumbar spine x-ray images according to a defined quality control standard.

**Materials and Methods:**

A dataset comprising anteroposterior, lateral, and oblique position lumbar spine x-ray images from 1,389 patients was analyzed in this study. The training set consisted of digital radiography images of 1,070 patients (800, 798, and 623 images of the anteroposterior, lateral, and oblique position, respectively) and the validation set included 319 patients (200, 205, and 156 images of the anteroposterior, lateral, and oblique position, respectively). The quality control standard for lumbar spine x-ray radiography in this study was defined using textbook guidelines of as a reference. An enhanced encoder-decoder fully convolutional network with U-net as the backbone was implemented to segment the anatomical structures in the x-ray images. The segmentations were used to build an automatic assessment method to detect unqualified images. The dice similarity coefficient was used to evaluate segmentation performance.

**Results:**

The dice similarity coefficient of the anteroposterior position images ranged from 0.82 to 0.96 (mean 0.91 ± 0.06); the dice similarity coefficient of the lateral position images ranged from 0.71 to 0.95 (mean 0.87 ± 0.10); the dice similarity coefficient of the oblique position images ranged from 0.66 to 0.93 (mean 0.80 ± 0.14). The accuracy, sensitivity, and specificity of the assessment method on the validation set were 0.971–0.990 (mean 0.98 ± 0.10), 0.714–0.933 (mean 0.86 ± 0.13), and 0.995–1.000 (mean 0.99 ± 0.12) for the three positions, respectively.

**Conclusion:**

This deep learning-based algorithm achieves accurate segmentation of lumbar spine x-ray images. It provides a reliable and efficient method to identify the shape of the lumbar spine while automatically determining the radiographic image quality.

## Introduction

Lower back pain (LBP) is a widespread public health problem and the main cause of disability worldwide (1–3). It is the sixth leading contributor to the global overall disease burden (4). LBP brings enormous economic and mental burdens to patients and has been found to reduce patient incomes by about 87% compared with the normal population (5).

At present, clinical lumbar X-ray is the preferred imaging method for the diagnosis of LBP; however, manual measurement of the various lumbar parameters is time-consuming, laborious, and inconsistent. Further, diagnosis based on lumbar spine x-ray images is greatly influenced by the physician's subjective perceptions.

Digital radiography (DR) is widely used in clinical practice due to its high image quality, low radiation dose, fast imaging speed, and simple operation process. In particular, lumbar DR can assist doctors to diagnose damage to the lumbar bone, such as tumors, bone tuberculosis, fractures, and deformity of the lumbar spine (6).

Despite the rapidity of lumbar DR, compared with magnetic resonance imaging (MRI) and computed tomography (CT), its effectiveness in radiologically suspicious examination of the lumbar spine is relatively poor. High-quality lumbar x-ray imaging can effectively avoid missed diagnosis and improve the accuracy of diagnosis (7). Routine radiography of the lumbar spine include the anteroposterior and lateral projection positions (8). Bilateral oblique (left and right oblique) radiography is taken as a supplement to routine radiographs to assist doctors in the diagnosis of lesions. The oblique position mainly captures an oblique view of the vertebral body, including the intervertebral joint space, and inferior and superior articular processes, and can be used to diagnose trauma, lumbar spondylolisthesis, inter-articular structure damage and crack, and vertebral facet joint lesions. However, technologists report that it is difficult to obtain the proper patient position in spinal examinations (9), leading to high rejection rates for spinal examinations generally. Therefore, the radiographic procedure for the lumbar spine needs to be standardized by medical guidelines (8, 10).

The quality of a medical image directly affects the diagnosis and treatment of the disease. Hence, a set of medical image quality standards have been established by experts based on domestic and foreign long-term clinical tests (11, 12). Poor radiographic operation skills, due to a lack of education and experience, result in poor quality DR images in clinical practice (9). Trained radiologists are considered to be the reference for task-based evaluation of medical image quality. However, the evaluation of a large number of images is time-consuming and error-prone (13). Therefore, realization of intelligent image quality control by a machine will have a greater auxiliary effect on radiographers' imaging work. Machine learning is used for medical image analysis to predict the disease curative effect and can also be used for image quality monitoring. Previously, an observer model was applied to optimize the parameters and evaluate the image quality of low-dose CT iterative reconstructions (14). Automated image quality evaluations using deep learning have also been performed for image quality evaluations of liver MRI (15).

Artificial intelligence (AI) has produced breakthroughs in medical imaging (16–18). By establishing support vector machines, convolutional neural network (CNN) models, and improved algorithms, the quantitative analysis and diagnosis of lumbar spine X-ray has been made possible. Li Y proposed a new neural network model based on feature fusion deep learning; this model combined shape and texture information of the lumbar spine to automatically locate and detect the vertebral body in lumbar X-ray images, without being impacted by metal fixation (19). Azimi developed a multi-variable model to predict recurrent lumbar disc herniation through multi-layer perceptron (20). Cho proposed a U-net framework that can quickly identify L1 (1st lumbar spine) and S1 (1st sacral vertebra) and can be used to evaluate lumbar lordosis (21). In 2020, Schwartz developed a CNN segmentation algorithm that was combined with a computer vision algorithm for the automatic measurement of scoliosis parameters from lumbar lateral X-ray images; this approach was found to have the potential to simplify the clinical workflow (22). The application of AI technology to medical imaging has excellent prospects for alleviating the workload of clinicians, effectively reducing or eliminating manual measurement errors, and assisting clinicians to quantitatively evaluate spinal deformities and other diseases more objectively.

For patient dose reduction, computer based solutions were implemented by using Monte Carlo simulation to investigate the extent of the effect of collimation on the absorbed organ dose (23, 24). Optimization of radiation dose and image quality is an important aspect of quality assurance procedures. Klaus suggested that a quality system should be implemented globally to ensure a high standard of radiographs produced in chiropractic clinics (25).

In this study, the aim was to develop an intelligent quality control model of lumbar spine x-ray radiography using deep learning via a fully convolutional neural network, U-Net. Real-time and retrospective evaluation of the deep learning-based model was performed on the validation dataset. The dice similarity coefficient (DSC), accuracy, sensitivity, and specificity were computed according to the criteria of the defined quality control standard. The experimental results demonstrated that the proposed quality control framework can be applied to the routine workflow of a radiographer in order to improve the diagnostic accuracy and efficiency of the clinician.

## Materials and Methods

### Definition of Reference Standard and Subjective Evaluation

According to x-ray radiography regulations (8, 10), lumbar spine radiography includes three positions, the anteroposterior, lateral, and left and/or right oblique positions. Different positions can be used to observe different anatomical structures. The qualified indicators for each position are described below.

#### Standard Anteroposterior View Image

a. The entire lumbar spine should be visible, with the T11/T12 (the 11th and 12th thoracic vertebra) at the top, the sacral region at the bottom, and laterally, the transverse processes and the sacroiliac joint should be included.
b. The vertebral bodies are located at the center of the image, with symmetrical transverse processes and the pedicle and sacroiliac joints on both sides. The patient is not rotated, and the spinous process is in the middle.
c. The intervertebral joints are clearly visible, allowing a clear view of the lumbar vertebral bodies, pedicles, and facet joints. The image has sufficient penetration and contrast to show the trabecular and cortical bones.
d. There is no bilateral shadow on the upper and lower margins of the third lumbar spine.

**Figure 2A1** shows the standard anteroposterior view.

#### Standard Lateral View Image

a. The entire lumbar spine should be visible, with T11/T12 at the top and the 2nd sacral vertebra at the bottom. Posterior columns and spinous processes should be complete.
b. The sciatic notch, superior articular surface, and upper and lower endplates should be overlapped.
c. The image has sufficient penetration and contrast to show the trabecular and cortical bones.
d. There is no bilateral shadow on the upper and lower margins of the third lumbar spine (L3).

**Figure 2B1** shows the standard lateral view.

#### Standard Oblique View Image

**Figure 1:**
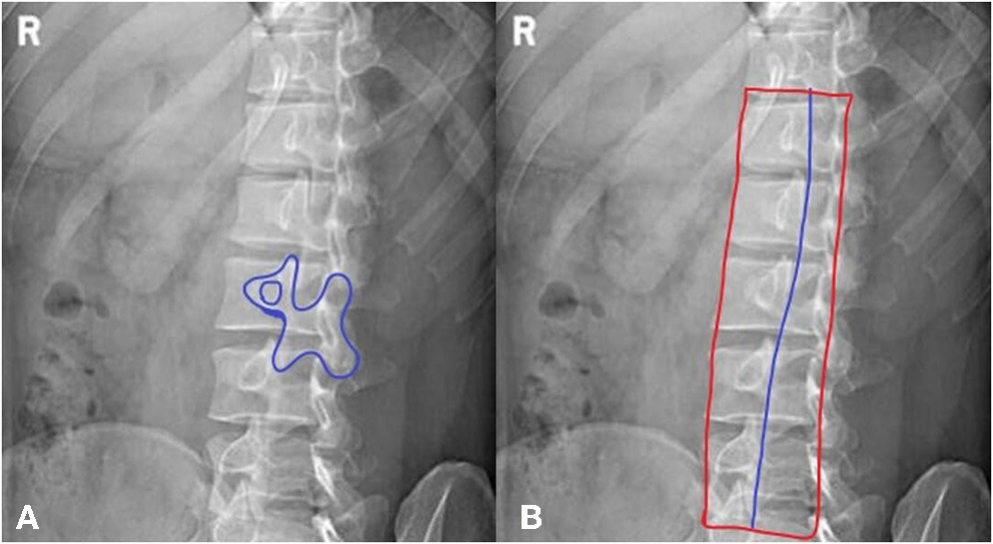
A method for jugement of “Dog” sign. **(A)** The dog's mouth is for ipsilateral transverse process. The dog's eye is for pedicle. The dog's ear is for superior articular process. The dog's neck is for interarticularis. The dog's body is for lamina. The dog's front leg is for inferior articular process. The dog's tail is for contralateral transverse process. **(B)** The inferior articular processes were connected in blue line.

a. The articular surfaces and joint spaces of the lumbar spine are clearly visible. The coverage is the same as the lateral image.
b. The “dog” sign is observed, showing the articular process and facet joints (as shown in Figure 1A).
c. The image has sufficient penetration and contrast to show the trabecular and cortical bones.
d. The pedicles are in the center of the vertebral body.

**Figure 2:**
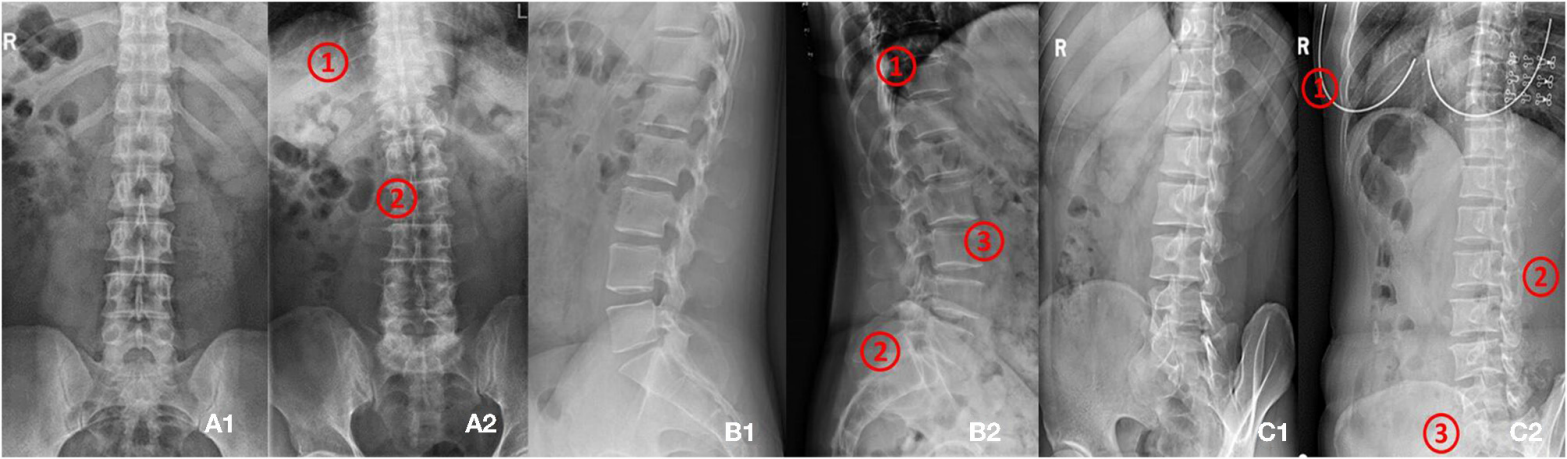
**(A1,A2)** Shows images of anteroposterior position. **(A1)** Shows qualified image. **(A2)** Shows unqualified image (1. Too many thoracics vertebrae; 2. Not centered and bent). **(B1,B2)** Shows images of lateral position. **(B1)** Shows qualified image. **(B2)** Shows unqualified image (1. Not clear; 2. Double shadow; 3. The left and right edges do not overlap). **(C1,C2)** Shows images of oblique position. **(C1)** Shows qualified image. **(C2)** Shows unqualified image (1. Excessive and foreign bodies in the chest; 2. Insufficient angle; 3. Less at the bottom).

Figure 2C1 shows the standard oblique view.

In this study, the lumbar spine x-ray images of 1,070 patients were evaluated by three experienced radiologists. The qualified x-ray images were those that met the criteria listed above for each radiograph position. Figure 2 presents examples of qualified and unqualified lumbar spine x-ray images.

### Datasets

**Figure 3:**
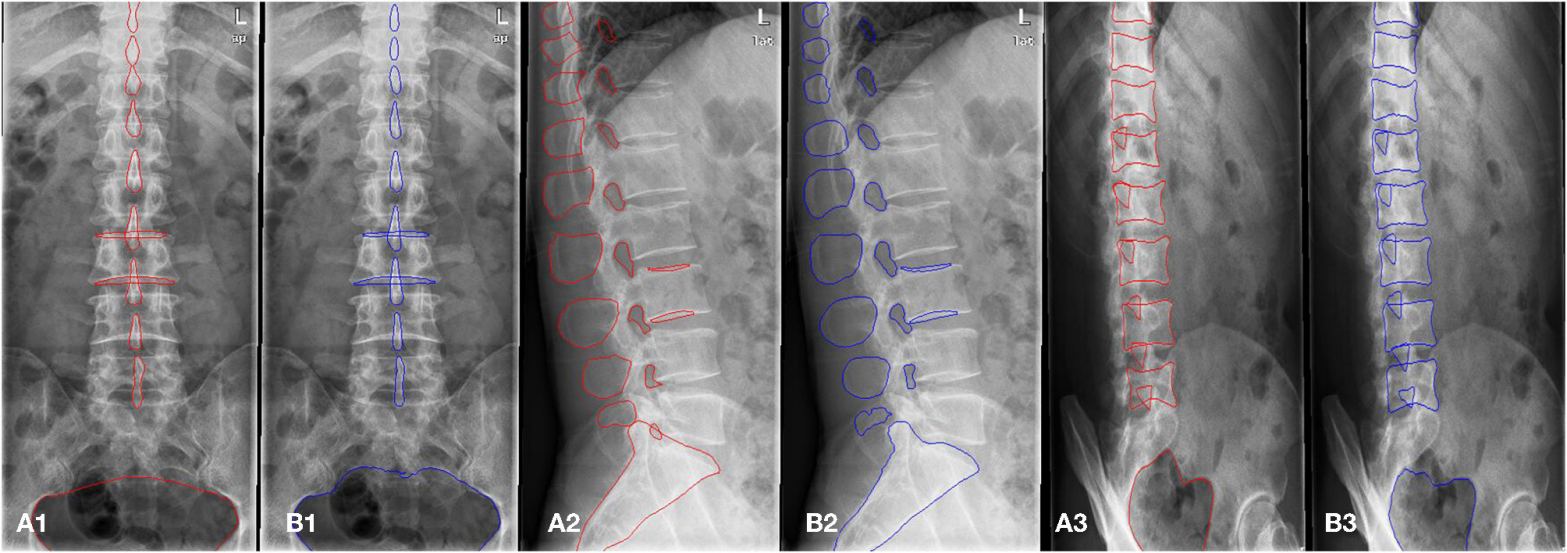
The example of manual segmentation and AI segmentation for three positions, anteroposterior **(A1,B1)**, lateral **(A2,B2)** and oblique view **(A3,B3)**. **(A1,A2,A3)** Ground truth of segmentation by manual marking. **(B1,B2,B3)** AI segmentation results.

A total of 1,389 patients were recruited into this cohort study by the First Affiliated Hospital of Wenzhou Medical University (Wenzhou, Zhejiang Province). Each patient had anteroposterior, lateral, and/or oblique position lumbar spine x-ray images taken. The dataset was randomly split into a training and a validation set. The training set consisted of 1,070 patients (800,798, and 623 images for the anteroposterior, lateral, and oblique position, respectively) and the validation set included 319 patients (200, 205, and 156 images for the anteroposterior, lateral, and oblique position, respectively). The regions of the lumbar vertebrae, pelvis, spinous processes, L3 (the third lumbar vertebra), and bilateral shadow of L3 were labeled on the fontal view of the lumbar spine, as shown in Figure 3A1. The area of the lumbar vertebrae, spinous processes, intervertebral foramen, L3, bilateral shadow of L3, and sacral vertebrae were marked on the lateral view of the lumbar spine, as shown in Figure 3A2. The lumbar vertebrae, inferior articular processes, and pelvis were marked on the oblique view of the lumbar spine, as shown in Figure 3A3. As ground truth for evaluation of the AI segmentation, all x-ray images were first delineated by two experienced radiologists, and then confirmed by a senior radiologist. This retrospective study was performed in accordance with the principles of the Helsinki Declaration and was approved by the institutional ethics committee. Given that this was a retrospective study, the need for obtaining written informed consent from the patients was waived.

### Image Segmentation Using Enhanced U-Net Architecture

**Figure 4:**
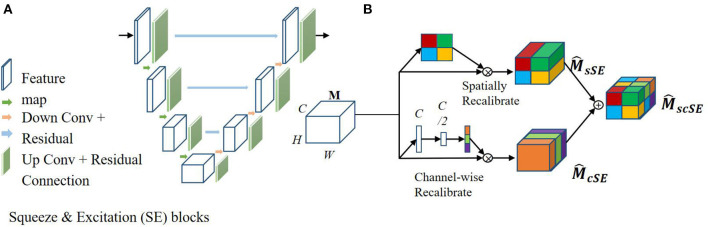
Architecture of the spatial information and channel Squeeze & Excitation “U-net.” The input of the network is the normalized image and the output is the probability map of the segmentation result. **(A)** SE blocks in U-net. **(B)** Spatial and channel SE block.

The fully convolutional neural network, U-net, is a state-of-the-art segmentation algorithm used in the medical image analysis field. Recent studies have focused on joint encoding of spatial and channel information to improve segmentation performance; however, joint encoding of the spatial and channel-wise information independently has been less studied. A squeeze & excitation (SE) block (26) has been proposed to address this gap by integrating spatial and channel dependencies. The SE block learns a channel-specific descriptor to recalibrate the feature map, and this is used to emphasize more important channels. In this work, the aim was to leverage the high performance of SE blocks for image segmentation with U-net, as shown in Figure 4A. In this current study, spatial and channel SE blocks (scSE) were implemented within the U-net frame, as shown in Figure 4B; the scSE blocks allowed for recalibration of the feature map along space and channel separately.

The scSE U-net model for image segmentation was applied to lumbar spine x-ray images taken from three positions, i.e., anteroposterior, lateral, and oblique. The first U-net was used to segment the anatomical features of the lumbar spine, such as the lumbar vertebra, pelvis, spinous process, intervertebral foramen, and sacral vertebrae. Then, within the obtained lumbar spine mask, the second U-net was used to automatically identify the anatomical features of the lumbar spine.

SE blocks can be inserted within a U-net model by integrating them after each encoder and decoder block, as shown in Figure 4A. Specifically, let us assume an output feature map *M*∈*R*^*H* × *W* × *C*^, here *H, W, and C* are the spatial height, width, and output channels, respectively. $\hat{M}$ is the recalibrated feature map by the SE blocks and is used in the subsequent pooling layers. In this work, two SE blocks were combined, a spatial SE block (spatial squeeze, sSE) and a channel SE block (channel squeeze, cSE); the combined SE blocks were denoted scSE block (27), as illustrated in Figure 4B. In the sSE block, the feature map is squeezed along the channels and excites spatially. For the cSE block, spatial squeeze is performed by a global average pooling layer. Finally, we can obtain ${\hat{M}}_{scSE}$ block, which is recalibrated spatially and channel-wise and is important for fine-grained image segmentation.

### Image Assessment Based on Defined Criteria

An automatic assessment system was developed based on the AI segmentation model. Image assessment was performed in reference to the criteria defined by textbooks (8, 10). These criteria were based on the important bone structures in the three x-ray image positions. For each criterion, quantitative measurement was performed based on manual scoring by experienced radiologists.

In terms of manual scoring, qualified images included the following elements: (a) seven spines (T11-L5) in every position, (b) in the anteroposterior position, the spinous process should be in the middle; there should be no bilateral shadow on the upper and lower margins of the third lumbar spine (L3) and the pelvis should be visible; (c) in the lateral position, there should be no bilateral shadow of L3, and the spinous process, intervertebral foramen, and sacral vertebrae should be visible; (d) in the oblique position, the “dog” sign (Figure 1A) should be observed and there should be at least three observed inferior articular processes; the pelvis should also be visible. A detailed description of this manual scoring is provided in Table 1.

**Table 1:** Criterion of objective and subjective evaluation.

	**Objective evaluation**	**Subjective evaluation**
**Anteroposterior position**		
Number of spines	7	7 (T11-L5)
Bilateral shadow/L3	(0, 0.21)	None
Position of spinous process	(0.4, 0.6)	Middle
Range of pelvis	>0	Visible
**Lateral position**		
Number of spines	7	7 (T11-L5)
Bilateral shadow/L3	(0, 0.21)	None
Spinous process	>0	Visible
Intervertebral foramen	>0	Visible
Sacral vertebrae	>0	Visible
**Oblique position**		
Number of spines	7	7 (T11-L5)
Range of pelvis	>0	Visible
Position of inferior articular processes	(0.265,0.365)	“Dog” signs: Observed (Number >3)

*The objective evaluations were based on the automatic segmentation results of the AI model*.

The automatic and quantitative measures were obtained based on the AI segmentation results. First, the visibility and number of the key anatomical features were computed directly from the segmented bone structures, such as the pelvis, intervertebral foramen, sacral vertebrae, and spinous process. Then, the number of segmented bone structures was used to assess whether the current x-ray image was qualified or not. For assessments based on the relative area and position, we learned from the ground truth in the training set and defined a series of ranges to determine the “dog” sign, bilateral shadow of L3 and the position of the spinous process. The objective criteria are listed in Table 1.

For instance, in the anteroposterior view of the lumbar spine, a centreline was created by connecting all centers of the segmented spinous processes. If the centreline lay within a certain range, defined by radiologists according to manual delineation, the current x-ray image was considered qualified.

For bilateral shadow, the areas of the bilateral shadow (A) and third lumbar vertebra (B) were calculated directly from the segmentation. The ratio of A to B was used to assess if there was obvious bilateral shadow.

In the oblique view of the lumbar spine, as shown in Figure 1B, the inferior articular processes of the lumbar vertebrae, which form the shape of a dog, were connected into a line. Their average position in the whole vertebrae was calculated. All qualified images that had the “dog” characteristic were counted and a certain range was obtained.

### Evaluation

#### Assessment of Segmentation Performance

The scSE U-net model was trained with 1,070 patients (800, 798, and 623 images for the anteroposterior, lateral, and oblique position, respectively) and then tested with the validation set (comprising 319 patients: 200, 205, and 156 images for the anteroposterior, lateral, and oblique position, respectively). The DSC was used to evaluate the segmentation performance. Figure 3 provides an example of manual segmentation and AI segmentation for the three positions.

All segmentation results were then used for the assessment of the qualification of x-ray images according to criteria defined in the textbooks (8, 10), as shown in Table 1. The performance of segmentation algorithms is crucial for x-ray image quality control. For anteroposterior position x-ray images, the outer contour, internal contour, pelvis, and spinous process were segmented by the U-net model. For lateral position x-ray images, segmentations included the outer contour, internal contour, intervertebral foramen, spinous process, and sacral vertebrae. For oblique position images, the inferior articular, vertebra, and pelvis were segmented from the x-ray images.

#### Evaluation of Image Quality Control

Ground truth was based on lumbar spine x-ray images collected from 1,389 patients and was determined by three experienced radiologists. Subjective and objective evaluations were carried out according to the defined criteria, as shown in Table 1. The objective evaluations were based on the automatic segmentation results of the AI model. An x-ray image was considered qualified when all objective criteria satisfied the thresholds, as listed in Table 1.

### Implementation

The proposed AI segmentation algorithms were implemented using Python (3.6) and Torch (1.2) with NVIDIA TITAN Xp graphic card (16 G Memory). All experiments are performed on Ubuntu system (16.04) computer with Intel E5-2620 CPU and 16 GB RAM.

## Results

### Segmentation Results

The DSC values for key features of lumbar spine radiographs based on the segmentation results of the AI model on the validation set are shown in Table 2. The segmentation performance in the anteroposterior position ranged from 0.82 to 0.96 (mean 0.91 ± 0.06), the performance in the lateral position ranged from 0.71 to 0.95 (mean 0.87 ± 0.10), and the DSCs in the oblique position ranged from 0.66 to 0.93 (mean 0.80 ± 0.14). The computation of the AI segmentation in anteroposterior, lateral, and oblique positions are 0.38, 0.37, and 0.34 s/frame, respectively.

**Table 2:** The segmentation performance (DSC value) of the scSE U-net model on the anatomy structures of anteroposterior, lateral, oblique position.

**Position**	**Segmented object**	**DSC value**
Anteroposterior position		
	Outer contour	0.923
	Internal contour	0.930
	Pelvis	0.960
	Spinous process	0.823
Lateral position		
	Outer contour	0.954
	Internal contour	0.935
	Intervertebral foramen	0.712
	Spinous process	0.816
	Sacral vertebrae	0.915
Oblique position		
	Inferior articular	0.655
	Vertebra	0.925
	Pelvis	0.829

### Classification Results

The final model obtained from the training set was applied to the validation set. The results of the automatic assessment are presented in Table 3. The thresholds for quantitative evaluation were based on manual scoring. In the anteroposterior position, the spinous processes were assessed to be in the middle of the spine when the average center points were between 0.4 and 0.6. In the anteroposterior and lateral position, if the bilateral shadow area/L3 area ratio was between 0 and 0.21, the third lumbar spine was assessed to have no bilateral shadow. In the oblique position, when the threshold of the position of the inferior articular processes was between 0.265 and 0.365, the “dog” sign was determined to be seen. For other key anatomical features, such as the pelvis, intervertebral foramen, and sacral vertebrae, the model segmentation was considered effective as long as the segmented part could be identified in the image. In each position, there should be seven visible spines, according to the criteria.

**Table 3:** Results of the automatic assessment system on the validation set that including 319 patients (200, 205, and 156 images for anteroposterior, lateral, and oblique position, respectively).

	**Ground truth**				
**AI Evaluation**	**Qualified**	**Unqualified**	**Accuracy (%)**	**Sensitivity (%)**	**Specificity (%)**
**Anteroposterior position**					
Qualified	13	1			
Unqualified	1	185			
Overall			99.0	92.9	99.5
**Lateral position**					
Qualified	28	4			
Unqualified	2	171			
Overall			97.1	93.3	97.7
**Oblique position**					
Qualified	5	0			
Unqualified	2	149			
Overall			98.7	71.4	100.0

**Figure 5:**
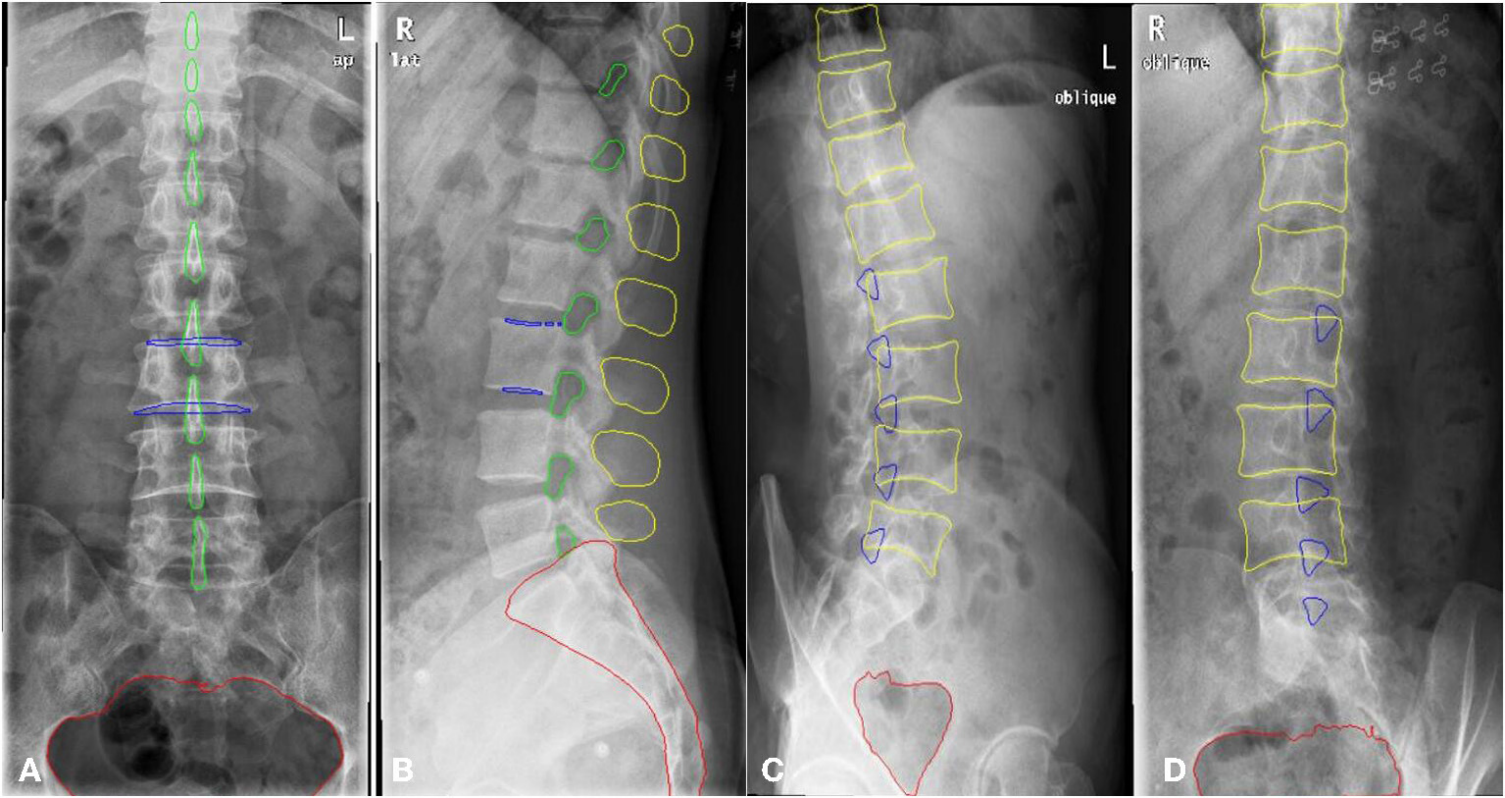
AI segmentation and automatic assessment by Quality Control Model. The unqualified cases **(A–C)**. The qualified case in **(D)**.

Figures 5A–C shows three unqualified x-ray images from the three different positions. In Figure 5A, it can be seen that there are more than seven spines. The assessment results for the bilateral shadow/L3 was 0.195215 and position of spinous process was 0.511964, which were in reasonable range.

In Figure 5B, the number of spines was greater than the threshold of seven. The assessment of the bilateral shadow/L3 was 0.071301, which was within the reasonable range. The intervertebral foramen and spinous processes were clearly visible. In Figure 5C, the number of spines was greater than seven. The value for the position of the inferior articular was <0.265, which means the “dog” sign was not visible. A qualified x-ray image is shown in Figure 5D. The number of spines was seven and the values of the positions of the inferior articular processes were in the right range, indicating that the “dog” sign was visible.

## Discussion

In this study, we developed a quality control model for lumbar spine radiography using a deep-learning method based on the U-net architecture (28) to automatically determine image quality. The model can accurately identify and segment the key anatomical features of the lumbar spine from three positions, and then quantitatively analyse the segmentation results. These quantitative indicators can be used for the determination of radiographic image quality based on radiographic standards; these standards are based on the reasonable threshold range defined based on manual analysis.

In the current study, the image quality analysis results of 319 patients in the validation set showed that the qualified rate was quite low for both the manual evaluation (7.0, 14.6, and 4.5% for the anteroposterior, lateral, and oblique position, respectively) and AI evaluation (7.0, 15.6, and 3.2%, respectively). These statistics indicate that image quality control management is crucial for lumbar radiography. Most of the unqualified images had more than seven spines, but the reasonable number of lumbar vertebrae is five according to normal physiological anatomy. This indicates that the photographic range was set too large during radiography. The aim of radiography is to obtain images that are adequate for clinical purposes with minimum radiation dose exposure to the patient. Therefore, the assessment of image quality is required to achieve a balance between the radiation dose and optimum performance. Reasonable control of the x-ray range can improve image resolution and effectively reduce radiation dose exposure to other parts of the patient, which is an essential aspect of the regulation of x-ray examinations (8).

Oblique radiography requires special attention as the patient's angle of tilt is critical to determining the presence of lesions at particular sites. The “dog's neck” represents the interarticularis. Clear visibility of the “dog” is essential to determining whether there is a break in the interarticularis. The threshold value in this article was defined by the position information of the inferior articular processes in the whole spines; this is an innovative method for judging the presence of the “dog” sign.

It is very difficult to identify the dog shape based on its features due to variation in patient body shape and position, and this study achieved good results based on quantitative assessments. For cases where the “dog” sign could manually be identified in the oblique position, the proposed model was able to accurately determine qualified images by the threshold value. This provides an innovative solution for x-ray image quality control.

**Figure 6:**
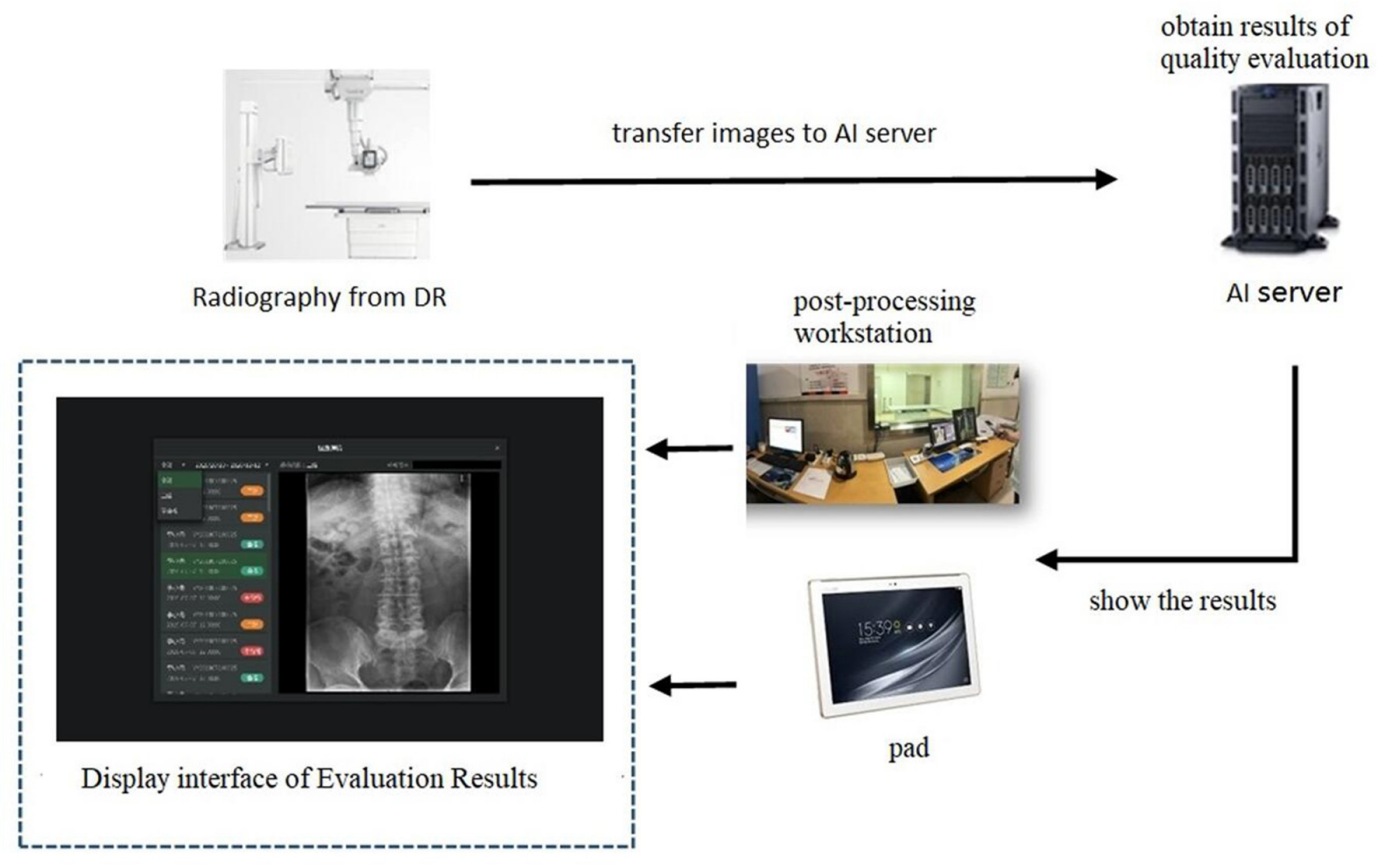
The application of Lumbar Spine X-ray radiography quality control model.

This model has significant application value due to its high accuracy in recognizing and segmenting the lumbar vertebrae. The following workflow is envisaged for applying this model (Figure 6). First, DR of the patient's lumbar spine is performed to obtain images of the anteroposterior, lateral, and oblique views. Second, the images are transferred to the server of the quality control model established by us, namely, the AI server. The model will detect, segment, and evaluate the x-ray images. Third, the evaluation results are presented on the post-processing workstation and can be displayed on a monitor or a small pad next to the DR device. Forth, based on the quality control evaluation results, the radiographers can check the images in real time. If unqualified images are found, the radiographers can re-photograph these views. For example, when photographing the lumbar oblique position, if the “dog” sign is not visible, the radiographer can adjust the angle of the patient's tilt and re-photograph to obtain a suitable diagnostic image. In addition, the quality control model can remind the radiographer to adjust the exposure range and avoid unnecessary radiation damage to the patient.

Radiographic image quality problems have increasingly been reported in recent years. Radiologists acknowledge that high-quality medical images contribute to the timeliness and accuracy of clinical diagnosis. Quality control in medical imaging is an ongoing process and not just a series of infrequent evaluations of medical imaging equipment. The quality control process involves designing and implementing a quality control program, collecting and analyzing data, investigating results that are outside the acceptance levels for the quality control program, and taking corrective action to bring these results back to an acceptable level (11). For example, some studies (29, 30) have improved the quality of thoracic CT examinations by providing patients with breathing training. The issues raised by Waaler and Hofmann (12) regarding the rejection and duplication of diagnostic x-ray images pose new challenges to radiographic imaging. The quality control process involves key personnel in the imaging department, including the radiologist, radiologic technologist, and qualified medical physicist.

Owusu-Banaheneaour's research (7) found that the highest rejection rate of 57.1 ± 0.7% was for cervical spine examinations in both adults and children, with overexposure and patient positioning being the actual causes; this is consistent with the current research. Obtaining high-quality radiographic images depends on quality control measures, proper selection of technical factors during exposure, and patient position. Shoulder and spinal examinations generally have the highest rejection rates. Technologists have reported that proper patient position can be difficult in these examinations (9). In the current study, the patient's position during lumbar oblique view radiography was critical to the imaging results. It was clear that there were significant differences in the way each technologist performed examinations and that some technologists were contributing to the overall rejection rate much more than others.

Recently, AI-based techniques have been applying to the control of medical image quality. Several intelligent AI models of chest radiographs have been published, and these can provide timely feedback on unqualified images. For example, in Hwang's and Annarumma's studies (31, 32), deep learning algorithms were used to classify the urgency of abnormal chest radiographs, so that abnormal cases could seek expert opinions as soon as possible. In one study, a CNN suitably adapted to the blind quality assessment task was found to accurately predict the quality of images with high agreement with human subjective scores (33). In another study, Alfaro-Almagro's research team developed an automated quality control tool to identify images with problems either in their acquisition or in the later processing steps (34). Tarroni proposed a fast, fully-automated, learning-based quality control pipeline for cardiac MR images, specifically for short-axis image stacks (35). Meineke suggested that machine learning can comprehensively detect CT examinations with dose optimization potential to simplify CT quality assurance (36).

Radiologists and radiographers are supportive of the application of AI technology in radiology practice (37). The study by Mohamed M found that radiographers' awareness of the role of AI and its challenges could be improved by education and training (38). Research also indicates that the practice of AI in radiology requires structured training programs for radiologists and radiographers in order to reduce work stress and better serve patients (38). The study by Abuzaid et al. (39) revealed that AI can be applied in MRI in various ways, such as to optimize image quality and avoid image artifacts.

The development of AI-assisted lumbar disease diagnosis is still in its early stages, and further exploration is needed to improve the AI algorithm and deep learning algorithm, establish a high-quality database, and formulate quantitative standards for new parameters.

Several limitations of this study should be noted. First, images with foreign objects, such as metal objects, were not segmented and detected. Although obvious foreign objects can be found in time, small external or internal foreign objects are easily overlooked. Secondly, variation in DR equipment can result in variation in image quality. This study did not detect the imaging quality of the machine itself, and thus, we cannot put forward suggestions for ideal resolution, signal-to-noise ratio, and other aspects. Further, this study was unable to assess whether the image quality was unqualified due to insufficient photographic conditions of the machine itself. Finally, the model described in this study has not been externally validated. The quality control model needs to be constantly updated to accommodate a variety of lumbar radiographs.

## Conclusion

In summary, we have developed a lumbar spine radiography quality control model based on U-net architecture. This model showed good segmentation accuracy and provided image quality evaluation results in real time. The proposed AI model allows for the standardization of radiographers' imaging work while reducing unnecessary radiation doses for patients.

## Data Availability Statement

The original contributions presented in the study are included in the article/supplementary material, further inquiries can be directed to the corresponding author/s.

## Ethics Statement

The studies involving human participants were reviewed and approved by Ethics Committee in Clinical Research (ECCR) of the First Affiliated Hospital of Wenzhou Medical University. Written informed consent for participation was not required for this study in accordance with the national legislation and the institutional requirements. Written informed consent was not obtained from the individual(s) for the publication of any potentially identifiable images or data included in this article.

## Author Contributions

XC, GC, and QW conceptualized and designed the study. GC and MW provided administrative support. QD, XL, and SL provided the study materials or patients. XC, QW, and XL collected and assembled the data. XC, QW, LC, and JL analyzed and interpreted the data. XC and LC wrote the manuscript. XC, QD, XL, LC, JL, QW, SL, MW, and GC gave the final approval of the manuscript. All authors contributed to the article and approved the submitted version.

## Funding

This research was supported by the grants from the National Key Research and Development Program of China (No. 2018YFC0116400), Wenzhou Municipal Science and Technology Bureau, China (Nos. Y20180185 and H2020002), Medical Health Science and Technology Project of Zhejiang Provincial Health Commission (No. 2019KY102), and Key Laboratory of Intelligent Medical Imaging of Wenzhou (No. 2021HZSY0057, Wenzhou, Zhejiang, China).

## Conflict of Interest

QW and LC were employed by Shanghai United Imaging Intelligence Co., Ltd. The remaining authors declare that the research was conducted in the absence of any commercial or financial relationships that could be construed as a potential conflict of interest.

## Publisher's Note

All claims expressed in this article are solely those of the authors and do not necessarily represent those of their affiliated organizations, or those of the publisher, the editors and the reviewers. Any product that may be evaluated in this article, or claim that may be made by its manufacturer, is not guaranteed or endorsed by the publisher.
